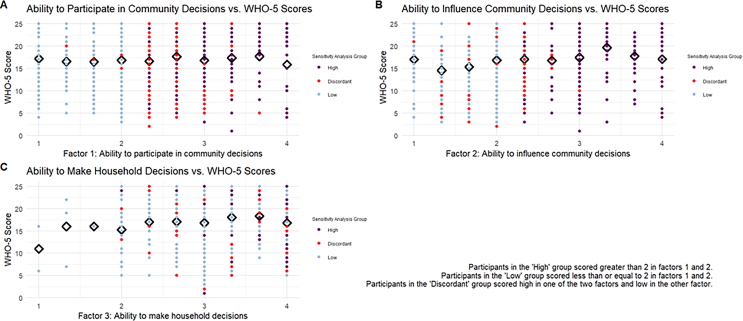# The association between women's decision-making roles in sanitation and mental well-being in urban Bangladesh

**DOI:** 10.1016/j.healthplace.2025.103515

**Published:** 2025-09

**Authors:** Nicole Stephan, Sheela S. Sinharoy, Rachel Waford, Madeleine Patrick, Thea Mink, Tanushree Bhan, Tanvir Ahmed, Alauddin Ahmed, Bethany A. Caruso

**Affiliations:** a Hubert Department of Global Health, Rollins School of Public Health, Emory University, Atlanta, GA, United States of America; b Department of Civil Engineering, Bangladesh University of Engineering and Technology, Dhaka, Bangladesh; c International Training Network, Bangladesh University of Engineering and Technology (ITN-BUET), Dhaka, Bangladesh

**Keywords:** Urban sanitation, Public health, Gender, Latrine access, Power dynamics

## Abstract

Research indicates that women and girls face gender-specific stressors related to sanitation that affect their mental health, and women's decision-making agency may play a role in this relationship. This study aimed to quantitatively assess the association between women's sanitation-related decision-making – overall, within the household, and within the community – and well-being in two urban municipalities in Bangladesh. This paper is a secondary analysis of cross-sectional survey data collected from 1449 women in Meherpur and Saidpur, Bangladesh from March–April 2022. We measured well-being using the World Health Organization Well-being Index [WHO-5] and primary exposures included women's sanitation-related decision-making and access to an unshared latrine. Decision-making was measured using the Agency, Resources, and Institutional Structures for Sanitation-related Empowerment (ARISE) scale; the full-scale score was utilized in the first analysis and the five individual factor scores were used in the second. Linear regression models were employed to assess the associations between decision-making and well-being scores, controlling for life stage, socioeconomic level, perceived social support and self-reported physical health. Analyses were conducted with city as a fixed effect and also stratified by city. Mean well-being scores were moderate in both cities, with approximately 20 % of respondents reporting poor well-being. On average, women generally agreed that they had a role in sanitation-related decision making (mean score = 2.66 on a 1–4 scale, with higher scores indicating stronger agreement that they had the ability to make sanitation-related decisions). In the full models using the full decision-making scale score, results indicated a positive association between the overall decision-making scale score and well-being (β = 0.73, p = 0.02). In full models using decision-making factor scores, we observed a positive association between well-being and individual decision-making factor scores for the ability to influence community-level sanitation decisions (β = 1.10, p = 0.0002) and to make small household sanitation decisions (β = 1.07, p = 0.006). Conversely, the ability to participate in community-level sanitation decisions was negatively associated with well-being (β = −0.68, p = 0.02). The ability to participate in household sanitation decisions and make large household sanitation decisions were not associated with well-being. Access to an unshared latrine was not associated with well-being in fully adjusted models. The relationships between decision-making factors and well-being varied in analyses stratified by city. Women's involvement in sanitation-related decision-making likely varies based on the extent of their participation, the nature of the decisions, and a range of contextual and household- or individual-level factors (e.g., socioeconomic level, age, education). To effectively improve women's well-being, sanitation programs must be informed by context-specific research exploring sanitation-related decision-making and its mediators among the populations of interest.

## Highlights

• Quantitatively assessed how women's sanitation-related decision-making relates to mental well-being in urban Bangladesh.
• Identified specific types of decision-making that are positively and negatively associated with women's well-being.
• Found that access to an unshared latrine alone was not associated with better mental health outcomes.
• Sanitation programs should ensure that women can meaningfully contribute to decision-making influence well-being.
• Highlighted the need for sanitation programs to move beyond infrastructure to support women's agency.

## 1 Introduction

Women and girls experience gender-specific health impacts related to inadequate sanitation, including adverse maternal health outcomes, altered eating or drinking habits to limit urination and defecation, and the inability to meet basic needs, including those related to menstrual health (Benova et al., 2014; Caruso et al., 2022; Caruso et al., 2017; Fewtrell et al., 2007; Fisher et al., 2017; Goddard and Marni, 2020; Khanna and Das, 2016; Padhi et al., 2015). Recognizing these disproportionate burdens, the 2030 Agenda for Sustainable Development aims to ‘achieve access to adequate and equitable sanitation and hygiene for all and end open defecation’ with ‘special attention to the needs of women and girls’ under Target 6.2. (United Nations, 2023; WHO/UNICEF Joint Monitoring Programme, 2023). Beyond physical health, a growing body of research shows that inadequate sanitation can cause women to face a greater burden of sanitation-related psychosocial stress – resulting in distress, anxiety, and poor well-being (Bisung and Elliott, 2017; Caruso et al., 2017; Hulland et al., 2015; Sahoo et al., 2015; Sclar et al., 2018). These stressors include shame from lack of privacy; physical or sexual violence; threat of injury from wildlife or the environment; and limited autonomy related to societal norms like caste, class, and gender. Research conducted in Odisha, India quantitatively demonstrated that sanitation insecurity (negative urination and defecation concerns and experiences) was linked to anxiety, depression, and poor well-being, irrespective of access to a functional household latrine (Caruso et al., 2018). Despite these findings, sanitation programs have historically focused on infrastructure development rather than prioritizing physical and social sanitation environments that are specifically designed to meet women's needs and reduce gendered stressors.

Various factors may influence the relationship between sanitation and mental health, beyond access to a functional latrine, including women's decision-making. It is hypothesized that improving decision-making influence grants women greater access to resources, ability to cope with stressful life circumstances, and overall self-efficacy (Richardson et al., 2019). Therefore, women's decision-making may contribute positively to their overall well-being (Richardson et al., 2019; Shankar et al., 2019). However, limited research has investigated women's sanitation-related decision-making and its relationship to well-being.

Current research shows that women typically have a limited role in sanitation-related decision-making, which negatively impacts their sanitation experiences (Annan et al., 2021; Das and Tampubolon, 2022; Khanna and Das, 2016; Routray et al., 2017). Qualitative studies in India found men did not prioritize the gender-specific needs of women when deciding if and how to construct latrines, which often led to women's avoidance of these latrines due to privacy, dignity, and safety concerns (Khanna and Das, 2016; Routray et al., 2017). As such, women's ability to exert control over their sanitation locations' placement, design, and maintenance may influence well-being (Khanna and Das, 2016; Routray et al., 2017; Sahoo et al., 2015).

Bangladesh is an appropriate location for exploring the relationship between women's sanitation-related decision-making and well-being. While the WHO/UNICEF Joint Monitoring Programme for Water Supply, Sanitation and Hygiene (JMP) reported open defecation to be eliminated in urban areas of Bangladesh, only 26.6 % of urban households have access to basic sanitation services, and 34.7 % have access to limited sanitation facilities (Joint Monitoring Programme, 2022). Additionally, Bangladesh has a patriarchal culture that is largely influenced by traditional and religious beliefs, and men typically have the final say in decisions (Chandramohan et al., 2023; Haque et al., 2011). While we were not able to identify research on gender and sanitation-related decision-making in Bangladesh, women's decision-making capabilities related to water were found to be limited (Clement, 2012). There is a need to investigate women's sanitation-related decision-making and well-being as findings can inform how future public health sanitation interventions in Bangladesh are planned and implemented.

This study aims to (1) understand women's perceived decision-making capabilities related to sanitation in two urban areas of Bangladesh, (2) evaluate the association between the use of unshared latrines and mental well-being and (3) examine the association between sanitation-related decision-making and mental well-being, (4) explore how associations between sanitation-related decision-making and well-being may differ based on overall decision-making and individual decision-making factor scores, and (5) assess how associations vary by city.

## 2 Methods

### 2.1 Study design

This study is a secondary analysis of cross-sectional data from Bangladesh collected as part of the Measuring Urban Sanitation and Empowerment (MUSE) project. The MUSE project aimed to develop and validate quantitative scales that measure women's sanitation-related empowerment in urban settings of low-income and middle-income countries. The study resulted in a finalized tool, the Agency, Resources, and Institutional Structures for Sanitation-related Empowerment (ARISE) scales, that measures 16 subdomains of sanitation-related empowerment and is informed by Van Eerdwijk et al.'s model of women's empowerment (Sinharoy et al., 2023; Van Eerdewijk et al., 2017). The ARISE scales can be used to assess subdomains of women's sanitation-related empowerment, informing the design, prioritization, and evaluation of sanitation initiatives. This analysis uses the decision-making scale, which captures the extent to which “women influence and make decisions about sanitation inside and outside the home,” to assess the association between women's sanitation-related decision-making and well-being (Sinharoy et al., 2022).

### 2.2 Study population, setting, and sampling procedure

Eligible study participants were women aged 18 years or older who spoke fluent Bangla or English, lived in targeted Mahallas (neighborhoods), were mentally competent (able to demonstrate understanding of the study description and consent), had no hearing or speech impediments that could impact comprehension or participation, and did not have another member of the household participate.

Data collection was led by the International Training Network - Bangladesh University of Engineering and Technology (ITN-BUET), in collaboration with two partners, WaterAid and Practical Action. WaterAid and Practical Action were responsible for overseeing the sampling and data collection in Saidpur and Meherpur, respectively. These two cities were selected due to the cooperative nature of the local government bodies. The target sample size was 700 participants per city, calculated for the larger MUSE study based on guidance for scale validation (Sinharoy et al., 2022).

Sampling was conducted in the two urban areas as follows. Saidpur, a municipality of the Nilphamari District within the Rangpur division, is divided into 15 wards and 43 Mahallas. WaterAid divided all Mahallas into 4–6 blocks, depending on the size of the area, and one block was randomly selected by a field supervisor. A list of all households in the block with at least one eligible woman (i.e., 18 years old or older, fluent in Bangla, mentally competent, and a permanent resident) was compiled and used as a sampling frame. Enumerators sampled every second household via the random walk technique. Enumerators sought to collect about 16–17 samples from each Mahalla to obtain a statistically representative sample and collected responses from 729 women. Meherpur is a municipality located in Meherpur district within the Khulna Division. A sample of 34 Mahallas in slum, non-slum, and mixed-income neighborhoods was identified using the standard probability proportional to size method. Due to the varied sizes of the Mahallas, each was further divided into clusters of 150 households. One cluster was chosen from each Mahalla using simple random sampling. The first household with women aged 18 years and older was randomly chosen from each cluster, then enumerators surveyed every second household and collected responses from 720 women.

### 2.3 Data collection

Data were collected from March–April 2022 in both Saidpur and Meherpur. Local female enumerators received five days of training, including the purpose of the parent study, definitions of women's empowerment, research ethics and logistics, and interactive practice conducting the survey with time for feedback and questions. The enumerators administered the ARISE survey instrument to eligible women who consented to participate. The survey included all 16 ARISE scales and six indices, as well as modules on demographics; water and sanitation access and behaviors; menstruation; and items to assess scale validity. All survey data was collected using tablets and Kobo Toolbox software. A field supervisor provided day-to-day supervision and quality control, including checking for survey completeness.

### 2.4 Measures

#### 2.4.1 Outcomes

The primary outcome of this analysis is subjective well-being, operationalized using the World Health Organization Well-being Index (WHO-5). The WHO-5 has been validated across global settings, including Bangladesh (Bech, 2004; Faruk et al., 2021; Topp et al., 2015). Subjective well-being is a “state in which the individual realizes his or her own abilities, can cope with the normal stresses of life, can work productively and fruitfully, and is able to make a contribution to his or her community” (World Health Organization, 2004). The WHO-5 was included in the survey to validate one of the ARISE Scales (Health) (Sinharoy et al., 2023). The WHO-5 was selected as the outcome of interest because research has demonstrated that positive well-being is associated with better mental health in addition to longevity, lower morbidity, less pain, and better self-reported health (Diener et al., 2017; Friedman and Kern, 2014; Vázquez et al., 2009). As a result, the WHO set an objective to shift focus towards positive indicators of mental health, including well-being, and research that would investigate the necessary conditions and strategies for enabling positive mental health (World Health Organization, 2004).

The WHO-5 asks participants to rate how often they have related to the following five statements over the past 2 weeks: (1) ‘I have felt cheerful and in good spirits', (2) ‘I have felt calm and relaxed’, (3) ‘I have felt active and vigorous', (4) ‘I woke up feeling fresh and rested’ and (5) ‘My daily life has been filled with things that interest me.’ Each item is scored from 0 (none of the time) to 5 (all of the time), and total possible scores range from 0 to 25. Higher scores indicate better well-being with scores below 13 indicating poor well-being (WHO Collaborating Center for Mental Health - Psychiatric Research Unit, 1998).

#### 2.4.2 Primary exposures

The primary exposures of this research study were access to an unshared (non-public) latrine and women's perceived decision-making influence related to sanitation both at home and within the community (Caruso et al., 2018). We used the term ‘unshared latrines’ to signify latrines that are used by one household exclusively (even if the household is renting or otherwise does not officially own the latrine). This term differs from ‘private latrine’, which are latrines that enable a user to experience privacy (e.g., ability to feel free from being observed, overheard, or disturbed by others while using sanitation facilities) (Caruso et al., 2024). Access to an unshared latrine was assessed by asking participants if they shared their sanitation location with others outside of their household. Those that did not share the location with others or used a private stall/stance in a shared latrine block – a group of toilets or latrines used by multiple households) – were coded as 1 (unshared latrine). Those who did not use an unshared latrine were coded as 0 (shared/public latrine).

Women's perceived decision-making ability related to sanitation was assessed using the ARISE decision-making scale. The scale (Sinharoy et al., 2023) captures women's perceived ability to make and influence sanitation decisions using 13 questions (items) that together comprise five factors, or sub-constructs of decision-making: 1) ability to speak up in community-level sanitation decision-making; 2) ability to influence community-level sanitation decision-making; 3) ability to participate in household-level sanitation decision-making; 4) ability to make large household-level sanitation decisions; and 5) ability to make small household-level sanitation decisions (see Table 1). The scale was validated in this study population, as described elsewhere (Sinharoy et al., 2023), and additional information is available in a published user guide.

**Table 1:** Factors and items of decision-making subdomain of ARISE scale (Sinharoy et al., 2023).

Sanitation-Related Decision-Making Scale	
Definition: Women's perceived ability to influence and make decisions about sanitation inside and outside the home.	
Response Options: 01: Strongly Disagree; 02: Disagree; 03: Agree; 04: Strongly Agree	
Item ID	Item
**Factor 1:** Ability to speak up in community-level sanitation decision-making	

DM01	I would feel comfortable expressing my opinion about sanitation issues at a community meeting when men are present
DM02	If I spoke up in a community meeting about sanitation issues, it is likely that some others would listen
DM03	If I shared my opinion about sanitation issues with local leaders, NGOs, or government officials, it is likely that they would listen.

**Factor 2:** Ability to influence community-level sanitation decision-making	

DM04	If my community had a major decision to make about sanitation, such as constructing public toilets, I could influence that decision.
DM05	If my community had decisions to make about latrine/toilet repairs or enhancements, like new floor tiles, doors, locks, or lights, I could influence these decisions
DM06	If my community had decisions to make about maintenance or cleaning of latrines/toilets, I could influence those decisions.

**Factor 3:** Ability to participate in household-level sanitation decision-making	

DM07	If my household was making a decision about sanitation-related issues, I could be present for the discussion.
DM08	If my household was making a decision about sanitation-related issues, I would be welcome to participate in the discussion.
DM09	I would feel comfortable expressing my opinion about sanitation issues in household discussions.

**Factor 4:** Ability to make large household-level sanitation decisions	

DM10	If my household had a major decision to make about sanitation, such as construction or large repairs, I could independently make that decision.
DM11	If my household had decisions to make about latrine/toilet repairs or enhancements, like new floor tiles, doors, locks, or lights, I could independently make that decision.

**Factor 5:** Ability to make small household-level sanitation decisions	

DM12	If my household had decisions to make about small sanitation-related purchases, like soap toilet paper, etc., I could independently make those decisions.
DM13	I can independently make decisions about how my household will clean and maintain the sanitation environment/facility.

Each of the 13 items was scored based on the extent to which women agreed with the statement using the following response options: Strongly Disagree (1), Disagree (2), Agree (3), and Strongly Agree (4). Overall scale and factor scores were calculated as simple, unweighted averages across all items or items in that factor (informed by validation procedures conducted in the parent study which used model-based omega to determine the best method for calculating test scores), respectively, with total possible scores ranging from 1 to 4 (Sinharoy et al., 2023). Higher overall scores indicate greater sanitation-related decision-making ability, and higher factor scores indicate greater decision-making ability as specified by the respective factor.

#### 2.4.3 Covariates

Covariates shown to impact mental health outcomes, including life stage, socioeconomic status, perceived social support, and perception of physical health, were included in analyses (Caruso et al., 2018; Patel, 2005). Participants were categorized into one of four life stages, as defined in Caruso et al. (2018), based on marital status and age: (1) unmarried, age 49 or younger (2) married three years or less and age 49 or younger, (3) married over three years and age 49 or younger, and (4) over 49 years of age of any marital status. These life stage categories were selected to capture differences in sanitation experiences associated with age and marital status (Caruso et al., 2018). Previous research has shown that marital status, years of marriage, and age allow for varying degrees of mobility, resource control, social status, and physical ability – all of which may influence sanitation decision-making (Joshi et al., 2011; Routray et al., 2015; Medhi, 2002; Singh et al., 2013).

Socioeconomic status was assessed using the World Health Organization International Wealth Index (IWI) and categorized into quintiles (Smits and Steendijk, 2015). Perceived social support was measured using eight items from the Multidimensional Scale for Perceived Social Support (MSPSS), a 12-item scale that assesses perceived social support from family, friends, and significant others (Zimet et al., 1988). Informed by Mohanty et al. (2017) and consistent with other research on sanitation and mental health, four items related to support from a significant other were excluded, as unmarried women were unlikely to have a significant other (Caruso et al., 2018; Mohanty et al., 2017). The MSPSS scale was scored according to the following response options, ranging from Completely Disagree (1), Mildly Disagree (2), Neither Agree nor Disagree (3), Mildly Agree (4), and Completely Agree (5), with a total possible score ranging from 1 to 5. Higher scores on the MSPSS indicate greater levels of social support. Perception of physical health was measured using one item from the Patient-Reported Outcome Measurement Information System (PROMIS) global health subscale, which asked, “In general, how would you rate your physical health?” (Pilkonis et al., 2014). Response options included and were scored as: Excellent (1), Very Good (2), Good (3), Fair (4), and Poor (5).

To assess the internal consistency of instruments, McDonald's omega was calculated for each of the multi-item scales used in the study. Omega is a measure of reliability that ranges from 0 to 1, with higher values indicating greater internal consistency among the items in a scale. Omega values can be found for relevant instruments in Table 3, Table 4.

**Table 2:** Exposures assessed in each model, type of analysis for location, *and* sample.

**A. Privately-Owned Larine Models**		
A. Unshared latrine *(Full sample, city as fixed effect)*	As. Unshared latrine *(Stratified: Saidpur only)*	Am. Unshared latrine *(Stratified: Meherpur only)*
**B. Full Decision-Making Scale Score Models**		
B1. Unshared latrine + overall Decision-Making score *(Full sample, city as fixed effect)*	B1s. Unshared latrine + overall Decision-Making score *(Stratified: Saidpur only)*	B1m. Unshared latrine + overall Decision-Making score *(Stratified: Meherpur only)*
B2. Unshared latrine + overall Decision-Making score + covariates *(Full sample, city as fixed effect)*	B2s. Unshared latrine + overall Decision-Making score + covariates *(Stratified: Saidpur only)*	B2m. Unshared latrine + overall Decision-Making score + covariates *(Stratified: Meherpur only)*
**C. Decision-Making Factor Scores Models**		
C1. Unshared latrine + Decision-Making factor scores *(Full sample, city as fixed effect)*	C1s. Unshared latrine + Decision-Making factor scores *(Stratified: Saidpur only)*	C1m. Unshared latrine + Decision-Making factor scores *(Stratified: Meherpur only)*
C2. Unshared latrine + Decision-Making factor scores + covariates *(Full sample, city as fixed effect)*	C2s. Unshared latrine + Decision-Making factor scores + covariates *(Stratified: Saidpur only)*	C2m. Unshared latrine + Decision-Making factor scores + covariates *(Stratified: Meherpur only)*

**Table 3:** Demographic characteristics of participants, overall and by city (N = 1149).

**Item**	**All**		**Meherpur**		**Saidpur**	
**Number of Participants**	1449		720	49.7 %	729	50.3 %
**Age** mean (SD)	33.92	(9.85)	35.63	(10.18)	32.24	(9.21)
**Marital status**						
Single/Never married	97	6.69 %	17	2.36 %	80	10.97 %
Married	1279	88.27 %	655	90.97 %	624	85.60 %
Unmarried, living with partner	0	0.00 %	0	0.00 %	0	0.00 %
Divorced/separated	15	1.04 %	14	1.94 %	1	0.14 %
Widowed	58	4.00 %	34	4.72 %	25	3.29 %
**Age first married**	16.90	3.33	16.19	3.14	17.67	3.36
**Life Stage**						
Stage 1: Unmarried or living with partner & ≤49 years old	97	6.69 %	17	2.36 %	80	10.97 %
Stage 2: Married under 3 years & ≤49 years old	58	4.00 %	24	3.33 %	34	4.66 %
Stage 3: Married greater than 3 years & ≤49 years old	1179	81.37 %	603	83.75 %	576	79.01 %
Stage 4: Over 49 years old	115	7.94 %	76	10.56 %	39	5.35 %
**Education**						
Completed primary or less	485	33.63 %	234	32.82 %	251	34.43 %
Completed secondary	823	57.07 %	416	58.35 %	407	55.83 %
Completed post-secondary	134	9.29 %	63	8.84 %	71	9.74 %
**Socioeconomic Level = Wealth Quintiles**						
Highest	251	17.33 %	148	20.56 %	103	14.15 %
Fourth	236	16.30 %	129	17.92 %	107	14.70 %
Middle	289	19.96 %	151	20.97 %	138	18.96 %
Second	485	33.49 %	215	29.86 %	270	37.09 %
Lowest	187	12.91 %	77	10.69 %	110	15.11 %
**Income Generating Activities**						
Does not earn an income	1065	73.50 %	492	68.33 %	573	78.60 %
Earns an income	384	26.50 %	228	31.67 %	156	21.40 %
**Type of Housing**						
Single family home	1246	86.05 %	644	89.44 %	602	82.69 %
Apartment	10	0.69 %	9	1.25 %	1	0.14 %
Compound with shared living spaces	174	12.02 %	66	9.17 %	108	14.84 %
Other	18	1.24 %	1	0.14 %	17	2.34 %
**Household Size** mean (SD)	4.49	1.94	4.40	2.02	4.58	1.86
**Religion**						
Christian (Catholic or Protestant)	12	0.83 %	0	0.00 %	12	1.65 %
Muslim	1329	91.78 %	645	89.58 %	684	93.96 %
Hindu	106	7.32 %	75	10.42 %	31	4.26 %
Buddhist	1	0.07 %	0	0.00 %	1	0.14 %
**Physical Health**						
Excellent	44	3.04 %	15	2.08 %	29	3.98 %
Very Good	232	16.02 %	115	15.97 %	117	16.07 %
Good	573	39.57 %	308	42.78 %	265	36.40 %
Fair	571	39.43 %	260	36.11 %	311	42.72 %
Poor	28	1.93 %	22	3.06 %	6	0.82 %
**Access to Privately-Owned (Non-Shared, Non-Public) Latrine in Household**						
Unshared latrine	1268	87.51 %	600	83.33 %	668	91.63 %
Shared latrine	181	12.49 %	120	16.67 %	61	8.37 %
**Social Support (Potential and actual range = 0–4,***ω* = **0.59)**	2.99	0.65	2.95	0.68	3.04	0.61
**WHO-5 Well-Being (Potential and actual range = 0–25, *ω* = 0.91))**	16.91	5.30	15.86	5.84	17.95	4.48
**WHO-5 Score of < 13 – indicating poor well-being**	292	20.15 %	211	29.31 %	81	11.11 %

Data are number and percent or mean and standard deviation. 1. Age first married: 17 missing from Meherpur, 80 missing from Saidpur; 2. Education: 7 missing from Meherpur; 3. Socioeconomic quintile: 1 missing from Saidpur; 4. Type of housing: 1 missing from Saidpur; 5. Religion: 1 missing from Saidpur; 6. Perception of physical health: 1 missing from Saidpur; 7. Social support: 1 missing from Saidpur; 8. WHO-5: 1 missing from Saidpur.

**Table 4:** Decision-making scores overall and by latrine status, by city.

Decision-Making Score	Both Cities			Meherpur			Saidpur		
	All (n = 1448)	Unshared latrine (n = 1267)	Shared/Public Latrine (n = 181)	All (n = 720)	Unshared latrine (n = 600)	Shared/Public Latrine (n = 120)	All (n = 1448)	Unshared latrine (n = 667)	Shared/Public Latrine (n = 61)
Full Scale (Cronbach's alpha = 0.97)	2.66 (0.43)	2.67 (0.43)	2.59 (0.42)	2.72 (0.46)	2.73 (0.46)	2.65 (0.48)	2.60 (0.38)	2.61 (0.39)	2.46 (0.26)
Factor 1 (***ω* =** 0.98)	2.33 (0.64)	2.32 (0.64)	2.36 (0.65)	2.52 (0.65)	2.52 (0.64)	2.48 (0.70)	2.14 (0.57)	2.14 (0.58)	2.13 (0.45)
Factor 2 (***ω*** = 0.97)	2.16 (0.64)	2.16 (0.64)	2.11 (0.67)	2.25 (0.73)	2.26 (0.72)	2.17 (0.76)	2.07 (0.53)	2.08 (0.54)	1.99 (0.42)
Factor 3 (***ω*** = 0.94	3.10 (0.49)	3.11 (0.48)	3.00 (0.51)	3.10 (0.49)	3.11 (0.49)	3.06 (0.49)	3.09 (0.48)	3.11 (0.47)	2.90 (0.53)
Factor 4 (***ω*** = 0.91)	2.80 (0.66)	2.83 (0.65)	2.60 (0.67)	2.78 (0.69)	2.81 (0.68)	2.65 (0.73)	2.82 (0.62)	2.85 (0.62)	2.52 (0.54)
Factor 5 (***ω*** = 0.94)	3.11 (0.52)	3.12 (0.52)	2.98 (0.56)	3.09 (0.55)	3.11 (0.54)	3.02 (0.59)	3.12 (0.50)	3.14 (0.49)	2.92 (0.48)

Data are mean and standard deviation.

### 2.5 Analysis

The association between women's perceived decision-making influence and well-being was investigated using multiple linear regression models. Two subsets of analysis were conducted: (1) using the aggregate scale score of the decision-making subdomain (i.e., the mean of all factor scores) to assess the overall association between decision-making and well-being, and (2) using all five factor scores within the decision-making subdomain as independent variables. Including all factors as separate variables was done to investigate if the association between decision-making and well-being differed based on women's perceived decision-making abilities at home or in the community, which the individual factors capture. Each analytic subset controlled for city as a fixed effect, then analyses were stratified by city to determine how location influenced the relationships.

For each of these subsets, three types of models were run, informed by the approach in Caruso et al. (2018) (see Table 2). Type A assessed the association between well-being and access to an unshared latrine alone, providing a baseline understanding of the relationship between unshared latrines and well-being without considering other variables. Type B evaluated the association between women's perceived decision-making and well-being while accounting for access to an unshared latrine. Type C included access to an unshared latrine, perceived decision-making, and all covariates (life stage, socioeconomic level, perceived social support, and perception of physical health). This full model was designed to control for potential confounders and offer a more nuanced understanding of the relationship between decision-making and well-being by examining how these factors collectively influence well-being.

A sensitivity analysis was conducted to evaluate the robustness of our findings and to identify if decision-making factors were interrelated. Specifically, we assessed whether the ability to participate in community decision-making (Factor 1) and the ability to influence community decision-making (Factor 2) were correlated and then assessed their respective relationships with well-being. We created scatterplots to illustrate the relationships between factors 1 and 2 and well-being among individuals who scored high (>2) or low (≤2) on both factors, as well as those with discordant scores between the two factors. All descriptive statistics and regression models were conducted in SAS (Cary, NC, USA; version 9.4) and omega values and figures were generated in RStudio (version 2023.12.1.402).

### 2.6 Ethics

This study received ethics approval from the Institutional Review Board at Emory University (Atlanta, GA, USA; IRB00072840) and the INT-BUET Research Committee at ITN-BUET (Dhaka, Bangladesh; ITN/Research/General/1.003/2022/02). All participants provided oral and written consent in Bangla or English prior to participation.

## 3 Results

### 3.1 Sample size and demographic characteristics

Across the two cities, 3160 respondents were approached to be surveyed; 720 women in Meherpur and 729 women in Saidpur completed the survey to comprise a total sample size of 1449 (see Fig. 1). The remaining 1711 approached individuals were excluded due to reasons such as having no eligible woman in the household, household inaccessibility, refusal, lack of consent, ineligibility, and incomplete responses. No participants were excluded from the analysis due to missing data. The average age of participants was 33.92 ± 9.85 (range: 18–80) years old. The average age when first married was 16.90 ± 3.33 (range: 10–37), 1279 (88 %) participants were married, 957 (66 %) had higher than a primary level of education, and 1065 (74 %) earned an income. A majority of participants practiced Islam (n = 1329, 92 %). Further details about sociodemographic characteristics in Meherpur and Saidpur can be found in Table 3.

**Fig. 1:**
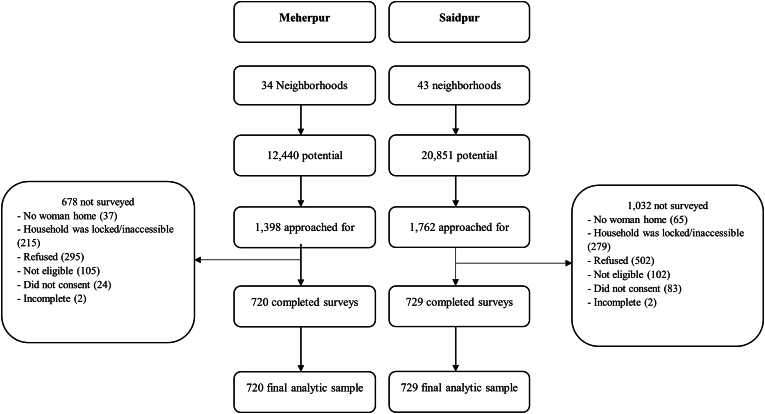
Flow diagram of recruitment, eligibility, survey completion, and final analytic samples by city.

59 % of participants rated their physical health as good or better (good: 40 %, very good: 16 %. excellent: 3 %), while 41 % indicated that their health was fair or poor. A majority of women (n = 1268, 88 %) had access to an unshared latrine, meaning they did not share their latrine with anyone outside of their household. More participants in Saidpur (n = 668, 92 %) had access to an unshared latrine than those in Meherpur (n = 600, 83 %).

#### 3.1.1 Descriptive results: decision-making and well-being scores

**Fig. 2:**
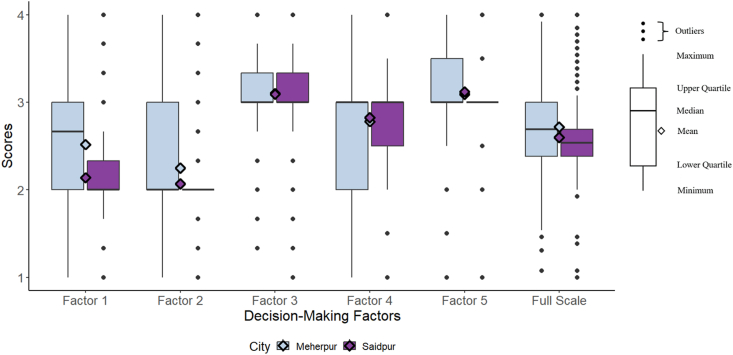
Box and whisker plot of decision-making scores by city.

The mean decision-making scale score for both cities was 2.66 out of a possible score of 4 (range = 1–4). This indicates that, overall, women slightly agreed that they have a role in sanitation-decision making but responses were mixed. Meherpur had a slightly higher mean decision-making score (mean = 2.72, range = 1.08–4) compared to Saidpur (mean = 2.60, range = 1–4). Of the five factors, the ability to influence community-level sanitation decision-making (Factor 2) (e.g., feel they have the ability to influence community decisions regarding latrine construction, repair, and maintenance) had the lowest overall factor score (mean = 2.16, range = 1–4). The ability to participate in household-level sanitation decision-making (Factor 3) (e.g., feel they are present for household discussions about sanitation and feel comfortable expressing their opinion) had the highest factor score (mean = 3.10, range = 1–4). The scores for the ability to participate in household-level sanitation decision-making (Factor 3) were comparable across cities. Women in Meherpur had higher scores for their perceived ability to speak up (Factor 1) and participate in community-level sanitation decision-making (Factor 2) while women in Saidpur had higher mean scores for the ability to make large (Factor 4) and small household-level sanitation decisions (Factor 5) (see Table 4). Overall, women with an unshared latrine had higher decision-making scores than those with a shared latrine for the full scale and all factors except the ability to participate in community-level decision-making (Factor 1), and there were larger differences between the two groups at the household level (see Table 4). The distribution of decision-making scores in Meherpur and Saidpur are shown in Fig. 2.

**Fig. 3:**
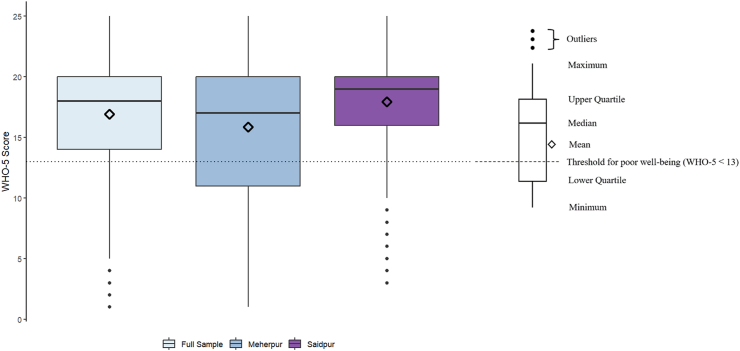
Box and whisker plot of well-being scores (WHO-5), overall and by city.

The average WHO-5 well-being score across cities was 16.91 (SD = 5.30, range = 1–25), indicating a moderate level of well-being overall (Topp et al., 2015). There was a higher proportion of women who scored below the threshold for poor well-being (a WHO-5 score of less than 13) in Meherpur (n = 211, 29 %) compared to Saidpur (n = 81, 11 %). Participants in Saidpur had slightly higher well-being scores (mean = 17.95, SD = 4.48, range = 3–25) compared to those in Meherpur (mean = 15.86, SD = 5.4, range = 1–25). Women who used unshared latrines had higher average well-being scores (mean = 17.13, SD = 5.21, range = 2–25) compared to those who used shared latrines (mean = 15.43, SD = 5.70, range = 1–25). Distributions of well-being scores by city can be found in Fig. 3.

### 3.2 Multivariate results

#### 3.2.1 Relationship between decision-making scale score and well-being

In models B1 and B2, we assessed relationships with the decision-making scale score, access to an unshared latrine, and well-being with city as a fixed effect. We found the decision-making scale score had a positive association with well-being in both the model excluding covariates (model B1) (β = 1.35, p < 0.001) and the fully adjusted model (model B2) (β = 0.73, p = 0.02) (see Table 5)—indicating that for each one-point increase in decision-making scores, there is a corresponding increase of 0.73 points in well-being scores.

**Table 5:** Association between access to an unshared latrine, sanitation-related decision-making scale score (aggregate score), individual covariates and well-being scores (WHO5) in Meherpur and Saidpur, Bangladesh. Full models. (Participants = 1448).

**Fixed Effects**												
Parameter Estimate, Standard Error, Confidence Interval, P-Value												
	**Access to an Unshared latrine**				**Access to an Unshared latrine and Aggregate Decision-Making Score**				**Access to an Unshared latrine, Aggregate Decision-Making Score, and Covariates**			
	**Model A1**				**Model B1**				**Model B2**			
**Intercept**	14.76	0.40	(13.98, 15.53)	<0.001^a^	11.21	0.93	(9.38, 13.04)	<0.0001^a^	18.05	1.25	(15.60,20.50)	0.0001^a^
**Access to an unshared latrine**	1.33	0.42	(0.51, 2.14)	0.001^a^	1.18	0.41	(0.37, 2.00)	0.004^a^	0.48	0.4	(-0.30,1.27)	0.23
**Decision-making Scale Score**					1.35	0.32	(0.72, 1.98)	<0.0001^a^	0.73	0.31	(0.12,1.33)	0.02^a^
**Life Stage**												
Stage 1: Unmarried or living with partner & ≤49 years old (referent)									–	–	–	–
Stage 2: Married under 3 years & ≤49 years old									0.16	0.81	(-1.42, 1.74)	0.84
Stage 3: Married greater than 3 years & ≤49 years old									−0.51	0.52	(-1.53, 0.51)	0.33
Stage 4: Over 49 years old									−1.39	0.68	(-2.73, −0.05)	0.04^a^
**Socioeconomic Level: Wealth Quintiles**												
Highest									2.49	0.49	(1.52, 3.45)	<0.0001^a^
Fourth									2.74	0.49	(1.78, 3.70)	<0.0001^a^
Middle									1.95	0.47	(1.03, 2.86)	<0.0001^a^
Second									1.5	0.42	(0.68, 2.32)	0.0004^a^
Lowest (referent)									–	–	–	–
**Physical Health**									−1.67	0.15	(-1.98, −1.37)	<0.0001^a^
**Perceived Social Support**									0.56	0.20	(0.16, 0.96)	0.01^a^
**Additional Model Components**												

R-Square	0.05				0.06				0.18			
F-value	34.40^a^				29.09^a^				26.45^a^			

a significant at p < 0.05.

Access to an unshared latrine was not associated with well-being in the full model (model B2; β = 0.48, p = 0.23), despite having positive associations with well-being in models A1 and B1. In the full model (model B2), all wealth quintiles were positively associated with well-being, with the second highest quintile having the strongest association (β = 2.74, p < 0.0001). Perceived physical health (β = −1.67, p < 0.0001) and a later life stage (stage 4: over age 49 years) (β = −1.39, p = 0.04) were negatively associated with well-being. As described above, a higher value for the physical health item indicates worse perceived health. Social support scores were positively associated with well-being (β = 0.56, p = 0.006).

Stratifying the data by city revealed that the overall decision-making scale score was no longer significantly associated with well-being (β = 0.66, p = 0.14) in Meherpur (model B2m), while in Saidpur, the positive association between decision-making and well-being remained (β = 1.11, p = 0.01) (model B2s). Additionally, older age (life stage 4: over age 49 years) was no longer associated with well-being (β = −0.83, p = 0.57) in Meherpur and social support was no longer positively associated with well-being in Saidpur (β = −0.23, p = 0.38). Additional results regarding models with the aggregate decision-making scale score stratified by city can be found in Appendices A, B, and C.

#### 3.2.2 Relationship between decision-making factor scores and well-being

In the full model with city as a fixed effect, including all decision-making factor scores, access to an unshared latrine, and well-being (model C2), two factors were positively associated with well-being: the ability to influence community-level sanitation decision-making (Factor 2) (β = 1.10, p = 0.0002) and the ability to make small household-level sanitation decisions (Factor 5) (β = 1.07, p = 0.006). Conversely, the ability to participate in community-level sanitation decision-making (Factor 1) was negatively associated with well-being (β = −0.68, p = 0.02). The remaining two decision-making factors (the ability to participate in household-level sanitation decision-making [Factor 3] and the ability to make large household-decisions [Factor 4]) were not associated with well-being (see Table 6). The sensitivity analysis conducted to determine if factors 1 and 2 were interrelated found no significant associations. Sensitivity analysis figures can be found in Appendix F and G.

**Table 6:** Association between access to an unshared latrine, sanitation-related decision-making (factor scores), individual covariates and well-being scores (WHO-5) in Meherpur and Saidpur, Bangladesh. Full models. (Participants = 1448).

**Fixed Effects**												
*Parameter Estimate, Standard Error, Confidence Interval, P-Value*												
	Model A1: Access to an Unshared latrine				Model C1: Access to an Unshared latrine and Decision-Making Factors				Model C2: Access to an Unshared latrine, Decision-Making Factors, and Covariates			
**Intercept**	14.76	0.40	(13.98, 15.53)	<0.001^a^	11.14	1.02	(9.14, 13.12)	<0.0001^a^	17.94	1.33	(15.33, 20.56)	<0.0001^a^
**Access to an unshared latrine**	1.33	0.42	(0.51, 2.14)	0.001^a^	1.20	0.41	(0.39, 2.01)	0.004^a^	0.46	0.40	(-0.33, 1.24)	0.25
**Decision-making**												
Factor 1: Ability to speak up in community-level sanitation decision-making					−0.28	0.32	(-0.90, 0.35)	0.38	−0.68	0.30	(-1.27, −0.09)	0.02^a^
Factor 2: Ability to influence community-level sanitation decision-making					1.10	0.31	(0.48, 1.71)	0.001^a^	1.10	0.29	(0.52, 1.67)	0.0002^a^
Factor 3: Ability to participate in household-level sanitation decision-making					−0.17	0.43	(-1.01, 0.67)	0.70	−0.40	0.40	(-1.18, 0.39)	0.32
Factor 4: Ability to make large household-level sanitation decisions					−0.50	0.27	(-1.03, 0.04)	0.07	−0.38	0.26	(-0.89, 0.12)	0.38
Factor 5: Ability to make small household-level sanitation decisions					1.25	0.41	(0.44, 2.05)	0.002^a^	1.08	0.39	(0.32, 1.84)	0.01^a^
**Life Stage**												
Stage 1: Unmarried or living with partner & ≤49 years old (referent)									–	–	–	–
Stage 2: Married under 3 years & ≤49 years old									0.03	0.80	(-1.55, 1.60)	0.97
Stage 3: Married greater than 3 years & ≤49 years old									−0.57	0.52	(-1.59, 0.45)	0.28
Stage 4: Over 49 years old									−1.31	0.68	(-2.65, 0.03)	0.06
**Socioeconomic Level: Wealth Quintiles**												
Highest									2.46	0.49	(1.50, 3.43)	<0.0001^a^
Fourth									2.76	0.49	(1.81, 3.72)	<0.0001^a^
Middle									1.95	0.47	(1.04, 2.86)	<0.0001^a^
Second									1.49	0.42	(0.23, 1.04)	0.0004^a^
Lowest (referent)									–	–	–	–
**Physical Health**									−1.67	0.15	(-1.98, −1.37)	<0.0001^a^
**Perceived Social Support**									0.63	0.21	(0.23, 1.04)	0.002^a^
**Additional Model Components**												

R-Square	0.05				0.07				0.19			
F-value	34.40^a^				14.79^a^				21.19^a^			

a significant at p < 0.05.

Access to an unshared latrine was initially positively associated with well-being when only including the decision-making factor scores in model C1 (β = 1.20, p = 0.004), but this relationship did not hold when all covariates were added (model C2; β = 0.45, p = 0.26). Similar to the results from the model B2, all wealth quintiles were positively associated with well-being and belonging to the second highest quintile had the strongest association (β = 2.76, p < 0.001). Perceived physical health was negatively associated with well-being (β = −1.67, <0.001) and social support was positively associated with well-being (β = 0.63, p = 0.002).

Differences in the relationships between decision-making factor scores and well-being emerged when the data were stratified by city. In Saidpur, the ability to participate in community-level sanitation decisions was positively associated with well-being (Factor 1) (β = 1.11, p = 0.01), while the ability to influence community-level sanitation decisions was negatively associated with well-being (Factor 2) (β = −0.91, p = 0.04) (model C2s). These relationships were reversed in Meherpur and in the overall dataset (see Table 7). Additionally, social support was no longer positively associated with well-being (β = −0.21, p = 0.45) and the second life stage (married under 3 years) was negatively associated with well-being (β = −2.12, p = 0.01). In Meherpur, the ability to make large household-level sanitation decisions was negatively associated with well-being (Factor 4) (β = −1.01, p = 0.01) (model C2m), while there was no association in Saidpur and the full sample. The positive association found between the ability to make small household-level decisions (Factor 5) and well-being in Saidpur and the full sample was no longer significant in Meherpur (β = 0.89, p = 0.16). Additional results for decision-making factor score models stratified by city can be found in Appendices D and E.

**Table 7:** Association between access to an unshared latrine, sanitation-related decision-making (factor scores), individual covariates and well-being scores (WHO-5) overall and by city. Full models*.*

Fixed Effects *Parameter Estimate, Standard Error, Confidence Interval, P-Value*														
	Model C2: Access to an Unshared latrine, Decision-Making Factors, and Covariates (All; n = 1448)				Model C2m: Access to an Unshared latrine, Decision-Making Factors, and Covariates (Meherpur; n = 720)				Model C2s: Access to an Unshared latrine, Decision-Making Factors, and Covariates (Saidpur; n = 728)					
**Intercept**	17.94	1.33	(15.33, 20.56)	<0.0001∗	14.97	2.39	(10.28, 19.65)	<0.0001∗	19.20	1.58	(16.10, 22.30)		<0.0001∗	
**Access to an unshared latrine**	0.46	0.40	(-0.33, 1.24)	0.25	0.64	0.55	(-0.44, 1.71)	0.25	0.21	0.58	(-0.93, 1.35)		0.72	
**Decision-making**														
Factor 1: Ability to speak up in community-level sanitation decision-making	−0.68	0.30	(-1.27, −0.09)	0.02^a^	−1.46	0.43	(-2.3, −0.61)	0.001^a^	1.11	0.43	(0.28, 1.95)		0.01^a^	
Factor 2: Ability to influence community-level sanitation decision-making	1.10	0.29	(0.52, 1.67)	0.0002^a^	2.14	0.40	(1.35, 2.92)	<0.0001^a^	−0.91	0.44	(-1.78, −0.03)		0.04^a^	
Factor 3: Ability to participate in household-level sanitation decision-making	−0.39	0.40	(-1.18, 0.40)	0.33	0.06	0.65	(-1.21, 1.33)	0.93	−0.92	0.48	(-1.87, 0.02)		0.05	
Factor 4: Ability to make large household-level sanitation decisions	−0.38	0.26	(-0.89, 0.12)	0.14	−1.01	0.41	(-1.82, −0.20)	0.01^a^	0.39	0.32	(0.25, 1.03)		0.23	
Factor 5: Ability to make small household-level sanitation decisions	1.07	0.39	(0.30, 1.83)	0.01^a^	0.89	0.62	(-0.34, 2.11)	0.16	1.37	0.49	(0.42, 2.32)		0.01^a^	
**Life Stage**														
Stage 1: Unmarried/living with a partner & ≤49 years old referent)	–	–	–	–	–	–	–	–	–	–	–		–	
Stage 2: Married under 3 years & ≤49 years old	0.03	0.80	(-1.55, 1.60)	0.97	1.07	1.72	(-2.30, 4.44)	0.53	−0.74	0.84	(-2.40, 0.91)		0.38	
Stage 3: Married >3 years & ≤49 years old	−0.57	0.52	(-1.59, 0.45)	0.28	−0.42	1.32	(-3.01, 2.16)	0.75	−0.89	0.50	(-1.88, 0.11)		0.08	
Stage 4: Over 49 years old	−1.31	0.68	(-2.65, 0.03)	0.06	−0.49	1.44	(-3.33, 2.35)	0.73	−2.12	0.81	(-3.70, −0.54)		0.01^a^	
**Socioeconomic Level: Wealth Quintiles**														
Highest	2.46	0.49	(1.50, 3.43)	<0.0001^a^	2.52	0.78	(0.99, 4.05)	0.001^a^	2.52	0.60	(1.34, 3.70)		<0.0001^a^	
Fourth	2.76	0.49	(1.81, 3.72)	<0.0001^a^	3.25	0.79	(1.70, 4.80)	<0.0001^a^	2.25	0.59	(1.09, 3.40)		0.0001^a^	
Middle	1.95	0.47	(1.04, 2.86)	<0.0001^a^	2.14	0.76	(0.65, 3.64)	0.01^a^	1.95	0.55	(0.86, 3.03)		0.001^a^	
Second	1.49	0.42	(0.23, 1.04)	0.0004^a^	1.95	0.72	(0.54, 3.36)	0.01^a^	1.22	0.47	(0.29, 2.15)		0.01^a^	
Lowest (referent)	–	–	–	–	–	–	–	–	–	–	–		–	
**Physical Health**	−1.67	0.15	(-1.98, −1.37)	<0.0001^a^	−1.87	0.25	(-2.37, −1.37)	<0.0001^a^	−1.40	0.18	(-1.76, −1.03)		<0.0001^a^	
**Perceived Social Support**	0.63	0.21	(0.23, 1.04)	0.002^a^	1.15	0.31	(0.55, 1.75)	0.0002^a^	−0.21	0.27	(-0.74, 0.33)		0.45	
**Additional Model Components**														

R-Square	0.19				0.19			0.19						
F-value	21.19^a^				10.62^a^				10.79^a^					

a significant at p < 0.05.

## 4 Discussion

We aimed to understand women's sanitation-related decision-making, the association between access to an unshared latrine, decision-making, and mental well-being, and how these associations differed between two cities in Bangladesh. Using the ARISE decision-making scale, we found that women in both cities slightly agree that they could have a role in sanitation-related decision-making. Our analysis revealed that access to an unshared latrine was not associated with well-being when adjusting for women's decision-making scores and other covariates. Yet, a higher overall perceived influence over sanitation-related decisions had a small but statistically significant association with higher subjective well-being. Furthermore, there was a more pronounced positive relationship between well-being and two subconstructs of decision-making: the ability to influence community-level sanitation decisions (Factor 2) and the ability to make small household sanitation purchases (Factor 5). One factor (Factor 1: the ability to participate in community-level sanitation decisions) had a significant negative association with well-being, while the remaining two decision-making factors (Factor 3: the ability to make household sanitation decisions overall and Factor 4: the ability to make large household sanitation decisions) had no associations with well-being. Lastly, we observed that the associations between decision-making scores and well-being varied by city.

Our findings that women's decision-making is associated with their well-being has important health implications for WASH programming. Within the SDGs, the United Nations has prioritized promoting well-being (goal 3), advancing gender equity and women's empowerment (goal 5), and ensuring adequate sanitation for all (goal 6) (UN General Assembly, 2015). Our research suggests that incorporating women's decision-making into sanitation interventions could advance progress towards each of these goals and aligns with findings from the WASH and women's empowerment literature (Caruso et al., 2018; Sahoo et al., 2015). Similarly, studies within the fields of women's agency and mental health have shown that decision-making is associated with lower rates of common mental disorders and mental distress (Das and Tampubolon, 2022; Patel et al., 2006; Richardson et al., 2019). The results of these studies taken together may suggest that increasing women's decision-making across many aspects of their lives, including sanitation, can have a meaningful impact on their mental health and well-being. Our work provides further evidence of the connection between women's sanitation-related experiences—specifically women's sanitation-related decision-making—and mental health and justifies additional study and evaluation of programs that investigate this relationship.

Our results align with other research that found access to sanitation facilities no longer has a significant relationship with well-being after controlling for sanitation-related experiences. Specifically, we found there to be no association between access to an unshared latrine and well-being once sanitation-related decision-making and covariates were included in models. A study conducted by Caruso et al. (2018) found that sanitation insecurity (negative urination and defecation concerns and experiences) was associated with negative mental health impacts across all outcomes regardless of household latrine access. Other studies have found that sanitation-related stressors, including financial stressors, physical access stressors (e.g., stress related to open defecation, injury or attacks from wildlife, and limited availability of latrines), and social stressors (e.g., lack of privacy, fear of violence, social conflicts over facilities, and shame or embarrassment related to latrines and open defecation) all contributed to negative mental health outcomes (Bisung and Elliott, 2017; Caruso et al., 2018; Sahoo et al., 2015; Sclar et al., 2018; Shiras et al., 2018). Our findings corroborate that access to a latrine alone does not mitigate the impact of negative sanitation experiences and stressors on well-being.

Looking at the individual factors of decision-making, the ability to participate in community meetings about sanitation was negatively associated with well-being, while the ability to influence community decisions was positively associated with well-being—findings which may explain the mixed results regarding the relationship between community participation and women's well-being from other studies. For example, a study conducted in Bangladesh found that women's ability to speak up in community spaces can positively influence well-being (Das and Tampubolon, 2022), while others in Bangladesh and India have indicated that this component of decision-making has a negative or neutral impact (Gruebner et al., 2012; Richardson et al., 2019). Furthermore, Bisung and Dickin (2021) argues that increasing women's participation in community decisions, without addressing the power dynamics between men and women, burdens women with additional time and labor constraints without offering any further sense of control. This argument aligns with qualitative work that has shown that women believe that they should have a say in community decisions, but often lack confidence when men are present or feel their opinions will not be acted upon due to existing gender norms (Clement, 2012; Doma et al., 2023; Kulkarni et al., 2017). Our results emphasize that initiatives that only aim to increase women's participation, without meaningfully increasing their influence, may be doing unintended harm.

Similarly, our results of a positive association between women's ability to make small household sanitation purchases and their subjective well-being contrasts with findings from other studies that showed household decision-making can negatively impact well-being (Hadley et al., 2010; Yount et al., 2014). These negative effects are hypothesized to arise when men and women's perceptions on women's autonomy disagree (Bisung and Dickin, 2021; Hadley et al., 2010; Richardson et al., 2019; Yount et al., 2014). Other studies in WASH and nutrition suggest that smaller sanitation-related decisions in the household may not contribute to additional intrahousehold conflict and resulting negative mental health outcomes. This idea is supported by qualitative research conducted in India, which found that women are expected to make smaller sanitation-related decisions (Doma et al., 2023). Other research in Bangladesh has found that women's perceived decision-making ability related to their own and their child's diet (another area where women are expected to make smaller decisions) was associated with greater self-efficacy (Salinger et al., 2024). Therefore, women's perceived ability to fulfill their assigned gender roles may contribute to a sense of well-being. At the same time, our results should not necessarily be seen as justification for assigning small sanitation-related decisions only to women. Rather, our results suggest that programs with a focus on increasing women's sanitation decision-making should first understand if and how shifts in decision-making may align or conflict with existing gender roles. If increasing women's decision-making may cause intrahousehold conflict, programs should seek to understand how to address household gender dynamics and norms. It may not be possible to change harmful gender norms, but initiatives should—at the very least—be careful not to strengthen them (Caruso et al., 2022).

The differences in the associations between decision-making factors and well-being seen between cities have important implications for public health practice and future research. These differences may stem from variations in demographic characteristics and contextual factors, such as cultural norms and community governance (Heintz et al., 2018). For example, more participants in Saidpur were in the earliest life stage (under 49 years old and unmarried) (Saidpur: 10.97 %, Meherpur: 2.36 %) whereas more participants in Meherpur fell into the latest life stage (over 49 years old) (Saidpur: 5.35 %, Meherpur: 10.56 %). A higher proportion of participants in Saidpur were in the lowest or second-lowest wealth quintile compared to those in Meherpur (Saidpur: 52.20 %, Meherpur: 40.55 %). The younger and more socioeconomically disadvantaged participants in Saidpur may have experienced a greater benefit to well-being from participation in community decisions but had less ability to influence community decisions—resulting in its negative association with well-being (Kirkwood et al., 2024). In addition, individuals at different life stages may have different expectations and support in both household and community level decision-making roles. It is also plausible that each city has different cultural values on community and household decision-making and community-based organization practices that can shape women's roles in decision-making. Further research from other cities and qualitative methods could clarify the underlying reasons for these differences. These findings emphasize that interventions must be tailored to the specific cultural context of the community.

The differences in decision-making scale scores between women with unshared latrines and those with shared latrines suggest varying decision-making contexts. However, the link between latrine ownership type and decision-making remains inconclusive. Studies conducted in India and Kenya found that women using shared latrines had lower decision-making power (Hirai et al., 2016; Sahoo et al., 2015). Additionally, households where women had more decision-making control over health and major household decisions were more likely to have improved latrines in their homes. Another study in coastal Odisha, India found no evidence that women's decision-making was higher in homes with an unshared latrine (Routray et al., 2017). It is possible that a combination of individual-level factors (e.g., education, age, financial resources) and household versus community-level decision-making dynamics contribute to the higher decision-making scores among those with unshared latrines. Women's ability to make decisions regarding sanitation at the community level may be limited by larger infrastructure factors, like operating and wait times and payment (Shiras et al., 2018). Furthermore, a study in Mozambique found that community-level decisions may be hindered by failures of collective action – or a group committing to preforming a set of actions and behaviors (Shiras et al., 2018). Women reported feeling unable to make meaningful changes to latrines because they were unable to influence others' behaviors regarding latrine management and maintenance. Although findings are mixed, they underscore that recommendations for improving women's sanitation decision-making must account for the context of the sanitation location. Interventions targeting unshared latrines may focus their efforts on household power-dynamics and norms about larger household financial decisions while those involving public latrines must address community collective action and gender-responsive infrastructure. (Hirai et al., 2016; Sahoo et al., 2015).

In the context of Bangladesh, our findings can inform how public health sanitation interventions are planned and implemented, while also addressing mental health needs. There is an opportunity to incorporate women's decision-making into sanitation programs, in ways that are contextually appropriate and do not cause harm by increasing intrahousehold conflict. Doing so can help advance women's empowerment and ensure that the needs of women and girls are met. Specifically, the WASH sector can strive to adopt gender transformative approaches, which seek to reconstruct gender norms and dynamics at the household, community, and institutional levels (as appropriate and practical) (MacArthur et al., 2023). WASH programs engage both men and women, integrate intersectional perspectives, and include gender-specific monitoring and evaluation to track gender equity progress and ensure long-term sustainability (Caruso et al., 2024; Caruso et al., 2022; MacArthur et al., 2023). Centering women's agency within the specific nature and context of their decision-making – without imposing Western-centric ideals in other cultural settings – is essential to ensure that gender-transformative efforts are truly empowering and contextually grounded. At the same time, we acknowledge that seeking to change power dynamics may be well beyond what WASH practitioners are able to do (or even what they should do), and that that attempts to change power dynamics can have negative consequences. At the very least, programs must prioritize harm reduction by not perpetuating or leveraging gender norms for programmatic gains, which many have done (Caruso et al., 2024). In some cases, WASH programs may find that incorporating gender responsive approaches, which incorporate gender dynamics into their work without explicitly attempting to dismantle structural barriers, are more appropriate based on cultural context and programmatic capacity.

Bangladesh has a limited mental healthcare infrastructure due to a lack of public mental health facilities, shortages of mental health professionals, insufficient mental healthcare funding, and societal stigma towards mental illness (Hasan et al., 2021a; 2021b; Islam and Biswas, 2014). Morbidity from mental illness remains high, and women in Bangladesh experience higher rates of mental illness compared to men but are half as likely to seek treatment (Hasan et al., 2021, Hasan et al., 2021; Koly et al., 2022). Our results demonstrate a statistically significant, though clinically modest, impact on women's well-being, suggesting that sanitation experiences are one of many factors that can be leveraged as a point of intervention to improve well-being. These findings contribute to the growing understanding of the relationship between sanitation and global mental health efforts. Global mental health and well-being is a critical public health issue that is necessary to fulfill the WHO's definition of health: “a state of complete physical, mental, and social well-being and not merely the absence of disease or infirmity,” (World Health Organization, 2004). Yet, mental disorders are one of the ten leading causes of global disease burden, and the number of disability-adjusted life years lost to mental disorders has increased from 80.8 million to 125.3 million since 1990 (GBD Mental Disorders Collaborators, 2022). To address the public health need associated with mental disorders, researchers argue that there is an urgent need to recognize and address the social determinants of mental health (SDOMH), which characterize the everyday impacts of social, economic, and political contexts on individuals' mental well-being (Burgess et al., 2020; Cosgrove et al., 2020; Patel et al., 2018). The SDOMH include factors from social and cultural, environmental, neighborhood, economic, and demographic domains, many of which interact and contribute to compounding effects on vulnerable populations (Patel et al., 2018). A number of studies have established the connection between inadequate sanitation and negative mental health (Bisung and Elliott, 2017; Caruso et al., 2018; Sahoo et al., 2015; Sclar et al., 2018). Our work further supports that sanitation-related experiences and specifically decision-making, as a component of the SDOMH, can serve as a point of intervention for public mental health efforts.

### 4.1 Strengths and limitations

This study contributes new evidence of the relationship between sanitation and women's mental health—specifically by exploring the association of women's sanitation-related decision-making and well-being. While there has been an increased interest in understanding how women's empowerment can be incorporated into WASH programming, few studies have investigated women's decision-making, and most have focused on water or failed to provide a clear definition of empowerment (Caruso et al., 2022). Our work utilized a validated, quantitative measurement of women's sanitation-related decision-making within a clearly defined empowerment framework and assessed decision-making beyond simply being present for decisions or participating in community meetings.

However, there are limitations to this study that warrant consideration when interpreting the findings. The study did not collect data on men's perspectives regarding women's decision-making ability, and, therefore, we were not able to assess if there was agreement between men and women within households and at the community level. Research has shown that improvements in women's well-being are strongest when men recognize women's empowerment. Collecting this data would allow further understanding of the relationship between decision-making and well-being (Bisung and Dickin, 2021). Future research efforts should include both men and women and investigate if the level of agreement between them and gendered power dynamics affects well-being outcomes. Additionally, unmeasured covariates, including previous life experiences, aspects of personality, and exposure to domestic violence, were not included in our data collection and models; these could confound the relationship between women's sanitation decision-making and well-being (Cheng and Furnham, 2001; Ellsberg et al., 2008; McEwen, 2003; Räikkönen and Pesonen, 2009; Steel et al., 2008) and could be explored in future research. Finally, our data are cross-sectional and do not allow the exploration of causal relationships. Future research should evaluate the causal effects of initiatives to improve women's sanitation-related decision-making on their well-being.

## 5 Conclusion

Women's decision-making related to sanitation overall has a positive association with well-being, but has differential effects, depending on the type of decisions being made and if they occur at the household or community level, within the urban municipalities of Meherpur and Saidpur, Bangladesh. In particular, women's ability to influence community decisions and contribute to household decisions about small purchases appeared to have the most beneficial impact on their well-being. Further research into the potential benefits of incorporating women's empowerment, specifically related to agency and decision-making, into WASH interventions is warranted and should be explored in other cultural and geographic contexts.

## CRediT authorship contribution statement

**Nicole Stephan:** Writing – review & editing, Visualization, Methodology, Conceptualization, Writing – original draft, Software, Formal analysis. **Sheela S. Sinharoy:** Writing – review & editing, Methodology, Funding acquisition, Project administration, Investigation, Conceptualization. **Rachel Waford:** Writing – review & editing, Supervision. **Madeleine Patrick:** Writing – review & editing. **Thea Mink:** Data curation, Writing – review & editing. **Tanushree Bhan:** Methodology, Writing – review & editing. **Tanvir Ahmed:** Project administration, Writing – review & editing. **Alauddin Ahmed:** Project administration, Writing – review & editing. **Bethany A. Caruso:** Writing – review & editing, Supervision, Conceptualization, Methodology.

## Figures and Tables

**figs1:**
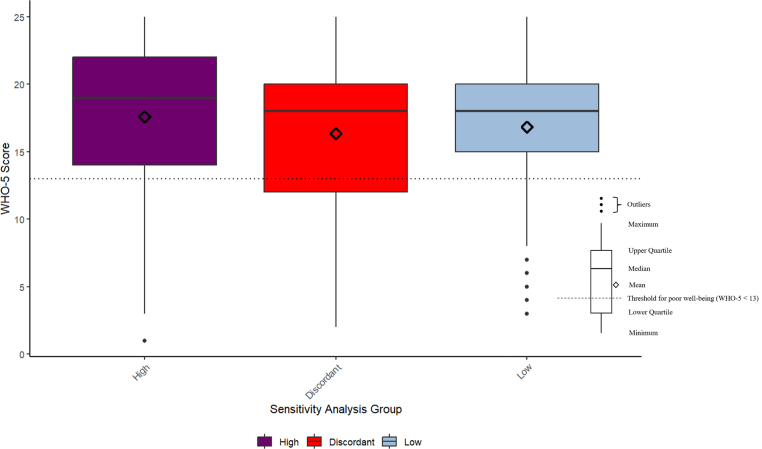


**figs2:**