# Supplementary material for: The association between women's decision-making roles in sanitation and mental well-being in urban Bangladesh
Source: Health Place. 2025 Sep;95:103515. doi: 10.1016/j.healthplace.2025.103515 (PMC12450114; doi:10.1016/j.healthplace.2025.103515)
Supplement: Multimedia component 1 [file mmc1.docx]

| ***Appendix A. Association between access to an unshared latrine, sanitation-related decision-making (aggregate score), individual covariates and well-being scores (WHO-5) in Meherpur, Bangladesh. Full models. (Participants=720)*** | | | | | | | | | | | | |
| --- | --- | --- | --- | --- | --- | --- | --- | --- | --- | --- | --- | --- |
|  | **Fixed Effects - Meherpur** | | | | | | | | | | | |
|  | *Parameter Estimate, Standard Error, Confidence Interval, P-Value* | | | | | | | | | | | |
|  | **Model A1m:**  **Access to an Unshared latrine** | | | | **Model B1m:**  **Access to an Unshared latrine and Aggregate Decision-Making Score** | | | | **Model B2m:**  **Access to an Unshared latrine, Aggregate Decision-Making Score, and Covariates** | | | |
| **Intercept** | 14.76 | 0.53 | (13.71, 15.80) | <.0001* | 12.24 | 1.35 | (9.58, 14.90) | <.0001* | 14.49 | 2.29 | (9.99, 18.99) | <.0001* |
| **Access to an unshared latrine** | 1.33 | 0.58 | (0.18, 2.47) | 0.02* | 1.25 | 0.58 | (0.11, 2.39) | 0.03* | 0.64 | 0.56 | (-0.45, 1.74) | 0.25 |
| **Decision-making Scale Score** |  |  |  |  | 0.95 | 0.47 | (0.03, 1.87) | 0.04* | 0.66 | 0.45 | (-0.22, 1.53) | 0.14 |
| **Life Stage** | | | | | | | |  |  |  |  |  |
| Stage 1: Unmarried or living with a partner & ≤49 year old (referent) | | | | | | | | | -- | -- | -- | -- |
| Stage 2: Married under 3 years & ≤49 years old | | | | |  |  |  |  | 1.37 | 1.74 | (-2.04, 4.78) | 0.43 |
| Stage 3: Married greater than 3 years & ≤49 years old | | | | |  |  |  |  | -0.45 | 1.34 | (-3.07, 2.18) | 0.74 |
| Stage 4: Over 49 years old | | | | |  |  |  |  | -0.83 | 1.47 | (-3.72, 2.05) | 0.57 |
| **Socioeconomic Level: Wealth Quintiles** | | | | |  |  |  |  |  |  |  |  |
| Highest |  |  |  |  |  |  |  |  | 2.45 | 0.79 | (0.90, 4.00) | 0.002* |
| Fourth |  |  |  |  |  |  |  |  | 3.15 | 0.80 | (1.58, 4.72) | <.0001* |
| Middle |  |  |  |  |  |  |  |  | 1.93 | 0.77 | (0.41, 3.45) | 0.01* |
| Second |  |  |  |  |  |  |  |  | 1.82 | 0.73 | (0.39, 3.24) | 0.01* |
| Lowest (referent) |  |  |  |  |  |  |  |  | -- | -- | -- | -- |
| **Physical Health** |  |  |  |  |  |  |  |  | -1.83 | 0.26 | (-2.33, -1.33) | <.0001* |
| **Perceived Social Support** | |  |  |  |  |  |  |  | 1.13 | 0.31 | (0.53, 1.74) | 0.0002* |
|  | **Additional Model Components** | | | | | | | | | | | |
| R-Square | 0.007 |  |  |  | 0.01 |  |  |  | 0.15 |  |  |  |
| F-value | 5.19* |  |  |  | 4.65* |  |  |  | 11.64* |  |  |  |

* significant at p<0.05
