# Supplementary material for: The association between women's decision-making roles in sanitation and mental well-being in urban Bangladesh
Source: Health Place. 2025 Sep;95:103515. doi: 10.1016/j.healthplace.2025.103515 (PMC12450114; doi:10.1016/j.healthplace.2025.103515)
Supplement: Multimedia component 2 [file mmc2.docx]

| ***Appendix B. Association between access to an unshared latrine, sanitation-related decision-making (aggregate score), individual covariates and well-being scores (WHO-5) in Saidpur, Bangladesh. Full models. (Participants=728)*** | | | | | | | | | | | | |
| --- | --- | --- | --- | --- | --- | --- | --- | --- | --- | --- | --- | --- |
|  | **Fixed Effects - Saidpur** | | | | | | | | | | | |
|  | *Parameter Estimate, Standard Error, Confidence Interval, P-Value* | | | | | | | | | | | |
|  | **Model A1s:**  **Access to an Unshared latrine** | | | | **Model B1s:**  **Access to an Unshared latrine and Aggregate Decision-Making Score** | | | | **Model B2s:**  **Access to an Unshared latrine, Aggregate Decision-Making Score, and Covariates** | | | |
| **Intercept** | 16.74 | 0.57 | (15.61, 17.86) | <.0001* | 11.97 | 1.20 | (9.61, 14.33) | <.0001* | 19.31 | 1.51 | (16.35, 22.26) | <.0001* |
| **Access to an unshared latrine** | 1.32 | 0.60 | (0.15, 2.50) | 0.03* | 1.03 | 0.59 | (-0.14, 2.19) | 0.08* | 0.16 | 0.58 | (-0.98, 1.30) | 0.78 |
| **Decision-making Scale Score** |  |  |  |  | 1.94 | 0.43 | (1.09, 2.79) | <.0001* | 1.11 | 0.43 | (0.26, 1.95) | 0.01* |
| **Life Stage** | | | | | | | |  |  |  |  |  |
| Stage 1: Unmarried or living with a partner & ≤49 years old (referent) | | | | | | | | | -- | -- | -- | -- |
| Stage 2: Married under 3 years & ≤49 years old | | | | |  |  |  |  | -0.66 | 0.85 | (-2.32, 1.01) | 0.44 |
| Stage 3: Married greater than 3 years & ≤49 years old | | | | |  |  |  |  | -0.65 | 0.50 | (-1.63, 0.34) | 0.20 |
| Stage 4: Over 49 years old | | | | |  |  |  |  | -1.92 | 0.81 | (-3.50, -0.33) | 0.02* |
| **Socioeconomic Level: Wealth Quintiles** | | | | |  |  |  |  |  |  |  |  |
| Highest |  |  |  |  |  |  |  |  | 2.74 | 0.60 | (1.56, 3.92) | <.0001* |
| Fourth |  |  |  |  |  |  |  |  | 2.39 | 0.58 | (1.24, 3.54) | <.0001* |
| Middle |  |  |  |  |  |  |  |  | 2.13 | 0.55 | (1.05, 3.21) | 0.0001* |
| Second |  |  |  |  |  |  |  |  | 1.30 | 0.47 | (0.37, 2.23) | 0.01* |
| Lowest (referent) |  |  |  |  |  |  |  |  | -- | -- | -- | -- |
| **Physical Health**  **Perceived Social Support** | | |  |  |  |  |  |  | -1.45 | 0.18 | (-1.81, -1.09) | <.0001* |
|  |  |  |  |  |  |  |  |  | -0.23 | 0.27 | (-0.76, 0.29) | 0.38 |
|  | **Additional Model Components** | | | | | | | | | | | |
| R-Square | 0.007 |  |  |  | 0.03 |  |  |  | 0.17 |  |  |  |
| F-value | 4.90* |  |  |  | 12.62* |  |  |  | 13.25* |  |  |  |

* significant at p<0.05
