# Supplementary material for: The association between women's decision-making roles in sanitation and mental well-being in urban Bangladesh
Source: Health Place. 2025 Sep;95:103515. doi: 10.1016/j.healthplace.2025.103515 (PMC12450114; doi:10.1016/j.healthplace.2025.103515)
Supplement: Multimedia component 3 [file mmc3.docx]

|  |  |  |  |  |  |  |  |  |  |  |  |  |
| --- | --- | --- | --- | --- | --- | --- | --- | --- | --- | --- | --- | --- |
| ***Appendix C. Association between access to an unshared latrine, sanitation-related decision-making (aggregate score), individual covariates and well-being scores (WHO-5) overall and by city. Full models.*** | | | | | | | | | | | | |
|  |  | **Fixed Effects**  *Parameter Estimate, Standard Error, Confidence Interval, P-Value* | | | | | | | | | | |
|  |  |  |  |  |  |  |  |  |  |  |  |  |
|  | **Model B2:**  **Access to an Unshared latrine, Decision-Making Score, and Covariates**  **(All; n=1448)** | | | | **Model B2m:**  **Access to an Unshared latrine, Decision-Making Score, and Covariates**  **(Meherpur; n=720)** | | | | **Model B2s:**  **Access to an Unshared latrine, Decision-Making Score, and Covariates**  **(Saidpur; n=728)** | | | |
|  |  |  |  |  |  |  |  |  |  |  |  |  |
| **Intercept** | 18.05 | 1.25 | (15.60, 20.50) | <0.0001* | 14.49 | 2.29 | (9.99, 18.99) | <.0001* | 19.31 | 1.51 | (16.35, 22.26) | <.0001* |
| **Access to an unshared latrine** | 0.48 | 0.40 | (-0.30, 1.27) | 0.23 | 0.64 | 0.56 | (-0.45, 1.74) | 0.25 | 0.16 | 0.58 | (-0.98, 1.30) | 0.78 |
| **Decision-making Scale Score** | 0.73 | 0.31 | (0.12, 1.33) | 0.02* | 0.66 | 0.45 | (-0.22, 1.53) | 0.14 | 1.11 | 0.43 | (0.26, 1.95) | 0.01* |
| **Life Stage** |  |  |  |  |  |  |  |  |  |  |  |  |
| Stage 1: Unmarried or living with a partner & ≤49 year old (referent) | -- | -- | -- | -- | -- | -- | -- | -- | -- | -- | -- | -- |
| Stage 2: Married under 3 years & ≤49 years old | 0.16 | 0.81 | (-1.42, 1.74) | 0.84 | 1.37 | 1.74 | (-2.04, 4.78) | 0.43 | -0.66 | 0.85 | (-2.32, 1.01) | 0.44 |
| Stage 3: Married greater than 3 years & ≤49 years old | -0.51 | 0.52 | (-1.53, 0 51) | 0.33 | -0.45 | 1.34 | (-3.07, 2.18) | 0.74 | -0.65 | 0.50 | (-1.63, 0.34) | 0.20 |
| Stage 4: Over 49 years old | -1.39 | 0.68 | (-273, -0.05) | 0.04* | -0.83 | 1.47 | (-3.72, 2.05) | 0.57 | -1.92 | 0.81 | (-3.50, -0.33) | 0.02* |
| **Socioeconomic Level:**  **Wealth Quintiles** |  |  |  |  |  |  |  |  |  |  |  |  |
| Highest | 2.49 | 0.49 | (1.52, 3.45) | <0 .0001* | 2.45 | 0.79 | (0.90, 4.00) | 0.002* | 2.74 | 0.60 | (1.56, 3.92) | <0.0001* |
| Fourth | 2.74 | 0.49 | (1.78, 3.70) | <0.0001* | 3.15 | 0.80 | (1.58, 4.72) | <.0001* | 2.39 | 0.58 | (1.24, 3.54) | <0.0001* |
| Middle | 1.95 | 0.47 | (1.03, 2.86) | <0.0001* | 1.93 | 0.77 | (0.41, 3.45) | 0.01* | 2.13 | 0.55 | (1.05, 3.21) | 0.0001* |
| Second | 1.50 | 0.42 | (0.68, 2.32) | 0.0004* | 1.82 | 0.73 | (0.39, 3.24) | 0.01* | 1.30 | 0.47 | (0.37, 2.23) | 0.01* |
| Lowest (referent) | -- | -- | -- | -- | -- | -- | -- | -- | -- | -- | -- | -- |
| **Physical Health** | -1.67 | 0.16 | (-1.97, -1.36) | 0.0001* | -1.83 | 0.26 | (-2.33, -1.33) | <0.0001* | -1.45 | 0.18 | (-1.81, -1.09) | <0.0001* |
| **Perceived Social Support** | 0.56 | 0.20 | (0.16, 0.96) | 0.01* | 1.13 | 0.31 | (0.53, 1.74) | 0.0002* | -0.23 | 0.27 | (-0.76, 0.29) | 0.38 |
| **Additional Model Components** | | | | | | | | | | | | |
| R-Square | 0.18 |  |  |  | 0.15 |  |  |  | 0.17 |  |  |  |
| F-value | 26.45* |  |  |  | 11.64* |  |  |  | 13.25* |  |  |  |

| * significant at p<0.05***Appendix D. Association between access to an unshared latrine, sanitation-related decision-making (factor scores), individual covariates and well-being scores (WHO-5) in Meherpur, Bangladesh. Full models. (Participants=720)*** | |
| --- | --- |
| * significant at p<0.05***Appendix D. Association between access to an unshared latrine, sanitation-related decision-making (factor scores), individual covariates and well-being scores (WHO-5) in Meherpur, Bangladesh. Full models. (Participants=720)*** | |

* significant at p<0.05
