# Supplementary material for: The association between women's decision-making roles in sanitation and mental well-being in urban Bangladesh
Source: Health Place. 2025 Sep;95:103515. doi: 10.1016/j.healthplace.2025.103515 (PMC12450114; doi:10.1016/j.healthplace.2025.103515)
Supplement: Multimedia component 4 [file mmc4.docx]

| ***Appendix D. Association between access to an unshared latrine, sanitation-related decision-making (factor scores), individual covariates and well-being scores (WHO-5) in Meherpur, Bangladesh. Full models. (Participants=720)*** | | | | | | | | | | | | |
| --- | --- | --- | --- | --- | --- | --- | --- | --- | --- | --- | --- | --- |
|  | **Fixed Effects - Meherpur** | | | | | | | | | | | |
|  | *Parameter Estimate, Standard Error, Confidence Interval, P-Value* | | | | | | | | | | | |
|  | **Model A1m:**  **Access to an Unshared latrine** | | | | **Model C1m:**  **Access to an Unshared latrine and Aggregate Decision-Making Score** | | | | **Model C2m:**  **Access to an Unshared latrine, Aggregate Decision-Making Score, and Covariates** | | | |
| **Intercept** | 14.76 | 0.53 | (13.71, 15.80) | <.0001 | 12.93 | 1.55 | (9.89, 15.96) | <.0001 | 14.97 | 2.39 | (10.28, 19.65) | <.0001 |
| **Access to an unshared latrine** | 1.33 | 0.58 | (0.18, 2.47) | 0.02 | 1.28 | 0.57 | (0.15, 2.40) | 0.03 | 0.64 | 0.55 | (-0.44, 1.71) | 0.25 |
| **Decision-making** |  |  |  |  |  |  |  |  |  |  |  |  |
| Factor 1: Ability to speak up in community-level sanitation decision-making | | | | | -1.29 | 0.46 | (-2.19, -0.39) | 0.01 | -1.46 | 0.43 | (-2.3, -0.61) | 0.001 |
| Factor 2: Ability to influence community-level sanitation decision-making | | | | | 2.27 | 0.43 | (1.43, 3.11) | <.0001 | 2.14 | 0.40 | (1.35, 2.92) | <.0001 |
| Factor 3: Ability to participate in household-level sanitation decision-making | | | | | 0.38 | 0.69 | (-0.97, 1.73) | 0.58 | 0.06 | 0.65 | (-1.21, 1.33) | 0.93 |
| Factor 4: Ability to make large household-level sanitation decisions | | | | | -0.99 | 0.44 | (-1.85, -0.13) | 0.02 | -1.01 | 0.41 | (-1.82, -0.20) | 0.01 |
| Factor 5: Ability to make small household-level sanitation decisions | | | | | 0.51 | 0.66 | (-0.79, 1.81) | 0.44 | 0.89 | 0.62 | (-0.34, 2.11) | 0.16 |
| **Life Stage** | | | | | | | |  |  |  |  |  |
| Stage 1: Unmarried or living with a partner & ≤ 49 years old (referent) | | | | | | | | | -- | -- | -- | -- |
| Stage 2: Married under 3 years & ≤49 years old | | | | |  |  |  |  | 1.07 | 1.72 | (-2.30, 4.44) | 0.53 |
| Stage 3: Married greater than 3 years & ≤49 years old | | | | |  |  |  |  | -0.42 | 1.32 | (-3.01, 2.16) | 0.75 |
| Stage 4: Over 49 years old | | | | |  |  |  |  | -0.49 | 1.44 | (-3.33, 2.35) | 0.73 |
| **Socioeconomic Level: Wealth Quintiles** | | | | |  |  |  |  |  |  |  |  |
| Highest |  |  |  |  |  |  |  |  | 2.52 | 0.78 | (0.99, 4.05) | 0.001 |
| Fourth |  |  |  |  |  |  |  |  | 3.25 | 0.79 | (1.70, 4.80) | <.0001 |
| Middle |  |  |  |  |  |  |  |  | 2.14 | 0.76 | (0.65, 3.64) | 0.01 |
| Second |  |  |  |  |  |  |  |  | 1.95 | 0.72 | (0.54, 3.36) | 0.01 |
| Lowest (referent) |  |  |  |  |  |  |  |  | -- | -- | -- | -- |
| **Physical Health** |  |  |  |  |  |  |  |  | -1.87 | 0.25 | (-2.37, -1.37) | <.0001 |
| **Perceived Social Support** | |  |  |  |  |  |  |  | 1.15 | 0.31 | (0.55, 1.75) | 0.0002 |
|  | **Additional Model Components** | | | | | | | | | | | |
| R-Square | 0.007 |  |  |  | 0.05 |  |  |  | 0.19 |  |  |  |
| F-value | 5.19* |  |  |  | 5.93* |  |  |  | 10.62* |  |  |  |

*Significant at p<0.05
