# Supplementary material for: The association between women's decision-making roles in sanitation and mental well-being in urban Bangladesh
Source: Health Place. 2025 Sep;95:103515. doi: 10.1016/j.healthplace.2025.103515 (PMC12450114; doi:10.1016/j.healthplace.2025.103515)
Supplement: Multimedia component 5 [file mmc5.docx]

| ***Appendix E. Association between access to an unshared latrine, sanitation-related decision-making scale score (factor scores), individual covariates and well-being scores (WHO-5) in Saidpur, Bangladesh. Full models. (Participants=728)*** | | | | | | | | | | | | |
| --- | --- | --- | --- | --- | --- | --- | --- | --- | --- | --- | --- | --- |
|  | **Fixed Effects - Saidpur** | | | | | | | | | | | |
|  | *Parameter Estimate, Standard Error, Confidence Interval, P-Value* | | | | | | | | | | | |
|  | **Model A1s:**  **Access to an Unshared latrine** | | | | **Model C1s:**  **Access to an Unshared latrine and Aggregate Decision-Making Score** | | | | **Model C2s:**  **Access to an Unshared latrine, Aggregate Decision-Making Score, and Covariates** | | | |
| **Intercept** | 16.74 | 0.57 | (15.61, 17.86) | <0.0001* | 11.69 | 1.25 | (9.23, 14.14) | <0.0001* | 19.20 | 1.58 | (16.10, 22.30) | <0.0001* |
| **Access to an unshared latrine** | 1.32 | 0.60 | (0.15, 2.50) | 0.03* | 1.08 | 0.59 | (-0.08, 2.24) | 0.07* | 0.21 | 0.58 | (-0.93, 1.35) | 0.72 |
| **Decision-making** |  |  |  |  |  |  |  |  |  |  |  |  |
| Factor 1: Ability to speak up in community-level sanitation decision-making | | | | | 1.59 | 0.44 | (0.72, 2.45) | 0.0003* | 1.11 | 0.43 | (0.28, 1.95) | 0.01* |
| Factor 2: Ability to influence community-level sanitation decision-making | | | | | -1.12 | 0.47 | (-2.04, -0.20) | 0.02* | -0.91 | 0.44 | (-1.78, -0.03) | 0.04* |
| Factor 3: Ability to participate in household-level sanitation decision-making | | | | | -0.80 | 0.51 | (-1.80, 0.21) | 0.12 | -0.92 | 0.48 | (-1.87, 0.02) | 0.05 |
| Factor 4: Ability to make large household-level sanitation decisions | | | | | 0.11 | 0.34 | (-0.55, 0.77) | 0.74 | 0.39 | 0.32 | (0.25, 1.03) | 0.23 |
| Factor 5: Ability to make small household-level sanitation decisions | | | | | 2.03 | 0.50 | (1.06, 3.01) | <0.0001* | 1.37 | 0.49 | (0.42, 2.32) | 0.01* |
| **Life Stage** | | | | | | | |  |  |  |  |  |
| Stage 1: Unmarried or living with a partner & ≤ 49 years old (referent) | | | | | | | | | -- | -- | -- | -- |
| Stage 2: Married under 3 years & ≤49 years old | | | | |  |  |  |  | -0.74 | 0.84 | (-2.40, 0.91) | 0.38 |
| Stage 3: Married greater than 3 years & ≤49 years old | | | | |  |  |  |  | -0.89 | 0.50 | (-1.88, 0.11) | 0.08 |
| Stage 4: Over 49 years old | | | | |  |  |  |  | -2.12 | 0.81 | (-3.70, -0.54) | 0.01* |
| **Socioeconomic Level: Wealth Quintiles** | | | | |  |  |  |  |  |  |  |  |
| Highest |  |  |  |  |  |  |  |  | 2.52 | 0.60 | (1.34, 3.70) | <0.0001* |
| Fourth |  |  |  |  |  |  |  |  | 2.25 | 0.59 | (1.09, 3.40) | 0.0001* |
| Middle |  |  |  |  |  |  |  |  | 1.95 | 0.55 | (0.86, 3.03) | 0.001* |
| Second |  |  |  |  |  |  |  |  | 1.22 | 0.47 | (0.29, 2.15) | 0.01* |
| Lowest (referent) |  |  |  |  |  |  |  |  | -- | -- | -- | -- |
| **Physical Health** |  |  |  |  |  |  |  |  | -1.40 | 0.18 | (-1.76, -1.03) | <0.0001* |
| **Perceived Social Support** | |  |  |  |  |  |  |  | -0.21 | 0.27 | (-0.74, 0.33) | 0.0002* |
|  | **Additional Model Components** | | | | | | | | | | | |
| R-Square | 0.007 |  |  |  | 0.06 |  |  |  | 0.19 |  |  |  |
| F-value | 4.90* |  |  |  | 8.15* |  |  |  | 10.79* |  |  |  |

* significant at p<0.05
